# Literary Note

**Published:** 1907-11

**Authors:** 


### LITERARY NOTE.

---

*The Hospital*, a high class weekly medical journal published in London, which was established twenty-one years ago, entered upon a new series with the issue dated April 6, 1907. It is now published as the "Modern" medical journal. The price of each number is 6 cents, and contains about 32 pages of literary matter.

In the makeup of the Hospital care is taken to include every modern development and all new discoveries, together with the methods of treatment and practice in this country, in the great European schools of medicine, and in the United States. The object is to make each section of the paper both compact and complete; and the whole is written with the desire to interest, help, and keep up to date practitioners engaged in the active service of their profession. We are glad to place *The Hospital* on our exchange list.

---

A LECTURE was given October 5, 1907, at the Library Building of the Medical Society of Kings County, in Brooklyn, by Dr. Thomas Darlington, President of the New York City Health Department, under the joint auspices of the Kings County Medical Society and the Brooklyn Institute. The attendance was so gratifying that Dr. Glentworth R. Butler, the president of the Medical Society, said, in introducing Dr. Darlington, that ample encouragement was afforded for the repetition of similar lectures on topics pertaining to the public health in the future. The subject of the evening was "Typhoid Fever and Other Infectious Diseases; How They are Spread."

